# Comparison of long-term outcomes of stereotactic body radiotherapy (SBRT) via Helical tomotherapy for early-stage lung cancer with or without pathological proof

**DOI:** 10.1186/s13014-023-02229-0

**Published:** 2023-03-08

**Authors:** Shaonan Fan, Qi Zhang, Jingyao Chen, Gang Chen, Jiangyi Zhu, Tingting Li, Han Xiao, Shisuo Du, Zhaochong Zeng, Jian He

**Affiliations:** Department of Radiation Oncology, Zhongshan Hospital, Fudan University, 180 Fenglin Road, Shanghai, 200032 China

**Keywords:** Stereotactic body radiotherapy, Helical tomotherapy, Early-stage lung cancer, Clinical diagnosis, Pathological diagnosis

## Abstract

**Background:**

Stereotactic body radio therapy (SBRT) has emerged as a standard treatment option for nonsurgical candidates with early-stage non-small cell lung cancer (NSCLC). Pathological proof is sometimes difficult to obtain in patients with solitary pulmonary nodules (SPNs). We aimed to compare the clinical outcomes of stereotactic body radiotherapy via helical tomotherapy (HT-SBRT) for early-stage lung cancer patients with or without a pathological diagnosis.

**Methods:**

Between June 2011 and December 2016, we treated 119 lung cancer patients with HT-SBRT, including 55 with a clinical diagnosis and 64 with a pathological diagnosis. Survival outcomes, including local control (LC), progression-free survival (PFS), cancer-specific survival (CSS), and overall survival (OS), were compared between two cohorts with and without a pathological diagnosis.

**Results:**

The median follow-up for the whole group was 69 months. Patients with a clinical diagnosis were significantly older (*p* = 0.002). No significant differences were observed between the clinical and pathological diagnosis cohorts in terms of the long-term outcome, with 5-year LC, PFS, CSS, and OS of 87% versus 83% (*p* = 0.58), 48% versus 45% (*p* = 0.82), 87% versus 84% (*p* = 0.65), and 60% versus 63% (*p* = 0.79), respectively. Recurrence patterns and toxicity were also similar.

**Conclusions:**

Empiric SBRT appears to be a safe and effective treatment option in a multidisciplinary setting when patients with SPNs highly suggestive of malignancy are unable/refuse to obtain a definitive pathological diagnosis.

**Supplementary Information:**

The online version contains supplementary material available at 10.1186/s13014-023-02229-0.

## Introduction

With the development and widespread use of medical screening tests such as low-dose computed tomography (CT) and fluorodeoxyglucose-positron emission tomography (FDG-PET), early-stage lung cancer is increasingly being discovered. Stereotactic body radiotherapy (SBRT), also known as stereotactic ablative radiotherapy (SABR), is currently the standard of care for patients with early-stage non-small cell lung cancer (ES-NSCLC) who are deemed medically inoperable [1–3], with an excellent rate of local control (LC) rate of approximately 90%, comparable to surgery [2]. Helical tomotherapy, which can deliver a conformal high dose of intensity-modulated radiation therapy (IMRT) to the target while minimizing the dose to surrounding organs at risk (OARs), is widely used in SBRT for ES-NSCLC [4, 5].

In principle, a definitive pathological diagnosis is essential for the treatment of primary lung cancer. However, a biopsy is not feasible for many inoperable patients because of the multiple comorbidities or wishes. In addition, even with repeat biopsies, pathological diagnosis is difficult due to tumor size and location. A systematic review showed that more than half of the published studies of lung SBRT had variable roportions of clinically diagnosed cases and up to 65% of patients are treated without a pathological diagnosis of malignancy [6].

Several studies have demonstrated the tolerability, feasibility, and efficacy of SBRT in patients with clinically diagnosed early-stage lung cancer, but these studies have lacked comparisons with contemporaneous pathologically diagnosed cohorts [7–10]. Few studies comparing clinical diagnosis with pathological diagnosis reported that survival outcomes appeared to be similar, although the median follow-up time in these studies was short (range 17–32.8 months) [11–14]. Although SBRT without pretreatment pathological confirmation has been utilized in patients with early-stage lung cancer, it remains controversial due to the limited long-term data on its efficacy.

In this study, we aimed to compare the long-term survival outcomes and toxicities of patients with or without pathological evidence of early-stage lung cancer patients treated with stereotactic body radiotherapy via helical tomotherapy (HT-SBRT).

## Materials and methods

### Patient selection

This study retrospectively reviewed the medical data of early-stage lung cancer patients treated with HT-SBRT at Zhongshan Hospital, Fudan University, between June 2011 and December 2016. Our multidisciplinary SPNs team, consisting of a radiologist, thoracic surgeon, pulmonologist, radiation oncologist, and pathologist, reviewed the clinical or pathological diagnoses and discussed treatment options for all patients. Tumors were staged according to the 8th edition of the American Joint Committee on Cancer (AJCC) TNM staging system [15]. All patients were discussed as either ineligible for surgery or refused surgery and were scheduled for SBRT in a multidisciplinary team (MDT). The data of FDG-PET were collected 1 month prior to SBRT. Exclusion criteria were as follows: (1) tumors greater than 5 cm in the greatest dimension; (2) patients with pathological diagnosis or suspicion of small cell lung cancer (SCLC) or large cell neuroendocrine carcinoma (LCNEC); (3) positive or suspected regional lymph node metastases, mediastinal spread, or systemic metastases inferred by CT, PET-CT, or endobronchial ultrasound (EBUS); (4) Eastern Cooperative Oncology Group (ECOG) performance status > 2; (5) 1 month or less of follow-up; and (6) patients with missing treatment information. The flowchart of patient selection is presented in Additional file 1: Figure S1.

The probability of malignancy was calculated for each patient based on a combination of clinical and radiological variables including progastrin-releasing peptide (ProGRP), carcinoembryonic antigen (CEA), squamous cell carcinoma antigen (SCC), and cytokeratin 19 fragment (CYRFRA21-1), age, smoking status, nodule diameter, spiculation, and sex (Additional file 2: Table S1). The nodule risk model is described by the following equations: probability of malignancy = e^x^/(1 + e^x^), x  = − 5.6017 + (0.0264 × age) + (8.8539 × smoke status) + (0.1859 × nodule diameter) + (3.1865 × spiculation) + (-8.7109 × sex) + (-0.00001 × ProGRP) + (0.0057 × SCC) + (0.1686 × CRFRA21-1) + (-0.00311 × CEA). A calculated cancer probability of 0.94 or higher indicates the presence of lung cancer (high risk), while a probability < 0.22 indicates the absence of lung cancer (low risk).

Finally, 119 patients were included in the study. For each patient, medical records were reviewed to obtain the data about demographic, clinical, and follow-up information.

### SBRT treatment details

All SBRT treatments were performed using a Helical Tomotherapy (HT) Hi-Art Treatment System (Accuray, Madison, WI, USA). The HT-SBRT technique and treatment planning were performed as previously described according to our institutional protocol [16]. The gross tumor volume (GTV) was delineated as a lesion observed at the lung window level on the enhanced CT and/or FDG-PET. The clinical target volume was equal to gross tumor volume. The internal target volume (ITV) was contoured based on the extension of GTVs at the all phases (5 inspiratory, 5 expiratory, and 1 resting phase) of the respiratory cycle on the four-dimensional CT (4D-CT) (Siemens Somatom Sensation, Siemens Healthineers Corporation, Germany) scanning to include the full movement of the tumor. To compensate for the uncertainty in tumor position and changes in tumor motion caused by breathing, the planning target volume (PTV) was extended by a margin of 0.5 cm from the ITV. Cone beam CT was implemented before each treatment to confirm the position of the target was achieved. The main factors determining the dose/fractionation scheme were tumor location, tumor size, and lung function parameters. In general, a total dose of 50 Gy/5 fractions (biologically effective dose [BED] = 100 Gy) was delivered for patients with peripherally located tumors and 60 Gy/10 fractions (BED = 96 Gy) was delivered for patients with centrally located tumors or tumors with extensive adherence to the chest wall. Dose constraints for the OARs were implemented according to the experience of the Radiation Therapy Oncology Group (RTOG) 0236 guidelines [2].

### Follow-up

In principle, follow-up chest CT scans were performed after the completion of SBRT every 4 weeks for the first 6 months, every 3 to 6 months for the next 30 months, every 6 months for the next 2 years, and annually thereafter, unless the patient refused or other obstacles. Other follow-up items included interviews, laboratory data review, and B-ultrasonography of the abdomen. Brain magnetic resonance imaging (MRI), bone emission computed tomography (ECT), and FDG-PET were also scanned if needed.

The follow-up duration was measured from the first day of SBRT to death or the date of the last follow-up. Events of interest included local control (LC), progression-free survival (PFS), cancer-specific survival (CSS), and overall survival (OS), which were calculated from the starting day of SBRT until the occurrence of an event of interest, or death or last follow-up. The treatment-related toxicity was assessed according to the Common Terminology Criteria for Adverse Events (CTCAE), version 5.0 [17].

### Statistical analyses

Baseline patient characteristics and trends in rates of radiotherapy use over time between the two cohorts were compared using either t-test, chi-squared or Fisher’s exact test, as appropriate. Continuous variables are presented as median and range. Categorical variables are presented as frequencies and percentages. Differences in treatment outcomes, including LC, PFS, CSS and OS, were calculated using Kaplan-Meier survival curves with log-rank tests. Statistical analyses were performed with R statistical software, version 4.0.5. A *p* value less than 0.05 was considered statistically significant. Power and sample size calculations were performed using PASS software version 11.0 based on the Lakatos method.

## Results

### Patient and treatment characteristics

A total of 119 patients underwent HT-SBRT for early-stage lung cancer between 2011 and 2016; of whom 55 (46%) had no biopsy and 64 (54%) were pathologically diagnosed with NSCLC. Although there was no significant change in the utilization of the two types of diagnosis during the study period (*p* = 0.41), the use of the clinical diagnosis increased over the years (Additional file 3: Figure S2). Demographic and clinical characteristics are shown in Table 1. The median age of patients with a clinical diagnosis was significantly higher than that of patients with a pathological diagnosis (76 years vs. 72 years, *p* = 0.002). Otherwise, both cohorts were well balanced in terms of patient and clinical characteristics.

**Table 1:** Demographic and clinical characteristics

	Characteristics	Total (*N* = 119)	Clinical diagnosis (*N* = 55)	Pathological diagnosis (*N* = 64)	*P* value
Age (years)	Median (range)	73 (40–89)	76 (61–89)	72 (40–89)	0.002
Gender	Female	35 (29%)	16 (29%)	19 (30%)	1
	Male	84 (71%)	39 (71%)	45 (70%)	
Smoking status	Current	28 (23%)	12 (22%)	16 (25%)	0.47
	Former	46 (39%)	19 (34%)	27 (42%)	
	Never	45 (38%)	24 (44%)	21 (33%)	
COPD	No COPD	34 (28%)	16 (29%)	18 (28%)	0.82
	GOLD I	33 (28%)	13 (24%)	20 (31%)	
	GOLD II	40 (34%)	20 (36%)	20 (31%)	
	GOLD III	12 (10%)	6 (11%)	6 (9%)	
CCI	0	14 (12%)	7 (13%)	7 (11%)	0.75
	1–2	65 (54%)	28 (51%)	37 (58%)	
	3+	40 (34%)	20 (36%)	20 (31%)	
ECOG PS	0	6 (5%)	3 (6%)	3 (5%)	0.89
	1	78 (66%)	37 (67%)	41 (64%)	
	2	35 (29%)	15 (27%)	20 (31%)	
Reasons for SBRT	Medically inoperable	105 (88%)	52 (95%)	53 (83%)	0.09
	Refusal of surgery	14 (12%)	3 (5%)	11 (17%)	
Tumor size (mm)	Median (range)	23 (4–50)	21 (10–48)	23 (4–50)	0.29
cT stage	T1a	9 (8%)	5 (9%)	4 (6%)	0.89
	T1b	44 (37%)	21 (38%)	23 (36%)	
	T1c	48 (40%)	22 (40%)	26 (41%)	
	T2a	14 (12%)	6 (11%)	8 (12%)	
	T2b	4 (3%)	1 (2%)	3 (5%)	
Histology	Non-diagnostic or no biopsy	55 (46%)	55 (100%)	0 (0%)	N.A
	Adenocarcinoma	40 (34%)	0 (0%)	40 (62%)	
	Squamous Cell	23 (19%)	0 (0%)	23 (36%)	
	NSCLC, NOS	1 (1%)	0 (0%)	1 (2%)	
Tumor location	Peripheral	80 (67%)	38 (69%)	42 (66%)	0.84
	Central	39 (33%)	17 (31%)	22 (34%)	
Pre-SBRT SUVmax	Median (range)	6 (0.4–29.4)	6 (0.9–29.4)	7 (0.4–23.6)	0.13
CT-fingding	Solid	57 (48%)	24 (44%)	33 (52%)	0.50
	GGO*	62 (52%)	31 (56%)	31 (48%)	

The criteria used to define ground-glass opacity (GGO) were referred to the Fleischner Society Glossary of Terms for Thoracic Imaging [18].

COPD = chronic obstructive pulmonary disease, GOLD = Global Initiative for Chronic Obstructive Lung Disease, CCI = Charlson comorbidity index, ECOG PS = Eastern Cooperative Oncology Group performance status, N.A. = not applicable, SBRT = stereotactic body radiotherapy, NOS = not otherwise specified, SUVmax = maximal standardized uptake value, GGO = ground-glass opacity

All patients received HT-SBRT. Analysis of dosimetric parameters between the clinical and pathological diagnosis cohorts (Table 2) revealed a lower lung V30Gy (lung volume receiving 30 Gy or more) and median ITV for the clinical diagnosis cohorts, but the differences were not significant. Other treatment characteristics were similar between the two cohorts.

**Table 2:** Dosimetric parameters

Characteristics		Total (*N* = 119)	Clinical diagnosis (*N* = 55)	Pathological diagnosis (*N* = 64)	*P* value
Dose fractionation schemes	50 Gy in 5 fractions (BED_10_ = 100 Gy)	80 (67%)	38 (69%)	42 (66%)	0.84
	60 Gy in 10 fractions (BED_10_ = 96 Gy)	39 (33%)	17 (31%)	22 (34%)	
Lung dose parameters					
	V5 (%), median (range)	22 (6–52)	20 (8–52)	25 (6–50)	0.22
	V10 (%), median (range)	12 (3–42)	11 (4–33)	13 (3–42)	0.11
	V20 (%), median (range)	5 (1–22)	5 (3–17)	6 (1–22)	0.10
	V30 (%), median (range)	3 (1–16)	2 (1–12)	3 (1–16)	0.07
	Dmean (Gy), median (range)	3.8 (1.5–13.6)	3.7 (1.5–11.1)	4.2 (1.9–13.6)	0.43
Esophagus dose parameters	Dmax (Gy), median(range)	13.3 (3.8–53.4)	14.5 (6.3–52.4)	12.7 (3.8–53.4)	0.80
PTV (cm^3^), median (range)		26.8 (2.9–99.4)	22.6 (2.9–89.3)	31.2 (4.0–99.4)	0.06
ITV(cm^3^), median (range)		13.6 (1.7–95.4)	16.0 (2.7–95.4)	17.3 (1.7–92.5)	0.77

Gy = Gray, BED_10_ = biologically effective dose using a/b-ratio of 10 Gy, Dmean = mean dose, Vn = the percentage of organ volume receiving ≥ nGy, PTV = planning target volume, ITV = internal target volume

### Calculation of the probability of malignancy

After calculation of the nodule risk model described above, the mean probability of malignancy in the clinical diagnosis and pathological diagnosis cohorts was 91% (95% CI 89–93%) and 93% (95% CI 92–94%, *p* = 0.88), respectively (Additional file 4: Figure S3).

### Survival outcomes

The median follow-up, calculated using the inverse Kaplan-Meier method [19], was 69 months (range, 5 to 117 months) for the whole group, 71 months for patients with a clinical diagnosis, and 67 months for patients with a pathological diagnosis.

**Fig. 1:**
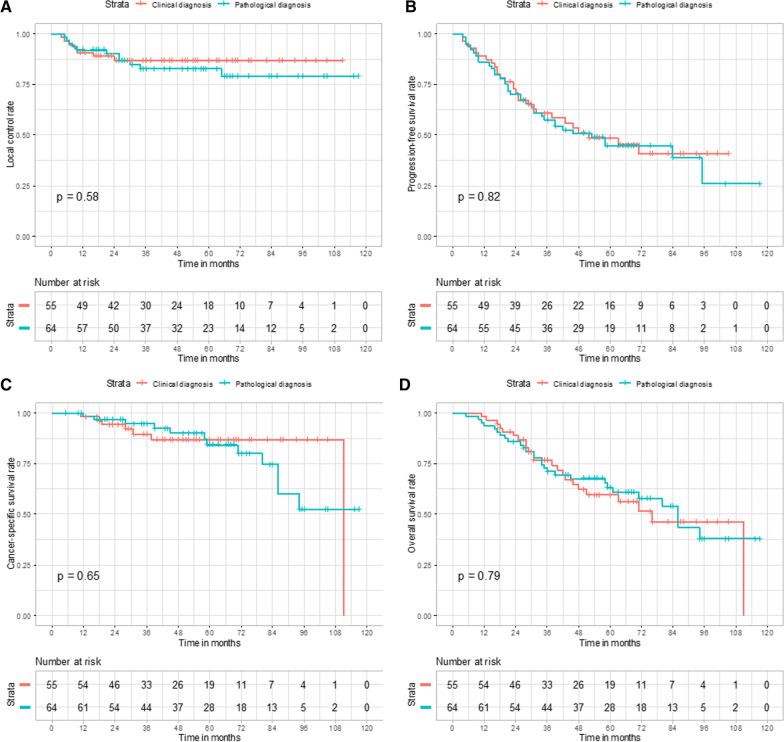
Comparison of local control (**a**), progression-free survival (**b**), cancer-specific survival (**c**), and overall survival (**d**) for patients with clinical versus patients with pathological diagnosis

Comparison of survival outcomes between the two patient cohorts is shown in Table 3 and Figures 1a–d. There was no significant difference in any of the outcome, with an estimated 5-year LC, PFS, CSS, and OS of 87% versus 83% (*p* = 0.58), 48% versus 45% (*p* = 0.82), 87% versus 84% (*p* = 0.65), and 60% versus 63% (*p* = 0.79), respectively.

**Table 3:** Survival outcomes

Outcomes	Clinical diagnosis (%)		Pathological diagnosis (%)		*P* value	Hazard ratio (95% CI)
	3-year	5-yarr	3-year	5-year		
Local control	87	87	83	83	0.58	0.77 (0.30–1.95)
Progression-free survival	61	48	58	45	0.82	0.94 (0.57–1.55)
Cancer-specific survival	90	87	95	84	0.65	0.81 (0.32–2.02)
Overall survival	77	60	71	63	0.79	1.08 (0.62–1.89)

CI = confidence interval

At the data cut-off point, 51 patients (43%) had died. There was no significant difference in cause of death between the clinical and pathological diagnosis cohorts, including lung cancer mortality (30% vs 43%, *p* = 0.56) and non-cancer mortality (52% vs 39%, *p* = 0.36) (Additional file 5: Table S2).

Forty-seven (40%) patients developed recurrence or metastasis. Intrapulmonary or mediastinal failure, the predominant pattern of failure (n = 30), occurred in 14 and 16 patients in the clinical and pathological diagnosis cohorts, respectively (Additional file 6: Fig. S4). Overall, the patterns of treatment failure did not differ between the two cohorts (Additional file 7: Table S3).

### Toxicity

No severe (CTCAE grade 4–5) toxicity was reported in either cohort. In total, five (4%) of the 119 patients experienced grade 3 acute radiation pneumonitis (RP). In the clinical diagnosis cohort, only one patient with a central tumor developed grade 3 acute radiation esophagitis (RE). No significant differences in toxicities were observed between the two cohorts (Table 4).

**Table 4:** Treatment toxicity

Events	Total (*N* = 119)	Clinical diagnosis (*N* = 55)	Pathological diagnosis (*N* = 64)	*P *value
Radiation pneumonitis (RP)				0.66
Grade 2	8 (7%)	4 (7%)	4 (6%)	
Grade 3	5 (4%)	3 (6%)	2 (3%)	
Radiation esophagitis (RE)				0.37
Grade 2	2 (2%)	1 (2%)	1 (2%)	
Grade 3	1 (1%)	1 (2%)	0 (0%)	
Chest wall toxicity (CWT)				0.37
G1	3 (3%)	1 (2%)	2 (3%)	

## Discussion

With the adoption of national lung cancer screening guidelines and advances in medical screening methods, an increasing number of SPNs are being diagnosed. The American College of Chest Physicians guidelines on the management of solitary pulmonary nodules recommend that non-surgical candidates with moderate to high clinical likelihood of malignancy or hypermetabolic nodules on FDG-PET imaging should undergo transthoracic needle biopsy or bronchoscopy to confirm a pathological diagnosis before further treatment [20]. However, even when small SPNs are highly suggestive of primary lung cancer, bronchial biopsy or CT-guided percutaneous lung biopsy sometimes fail to make a diagnosis because of tumor size and location, or patient refusal [10]. In addition, the presence of multiple comorbidities, such as COPD and cardiovascular disease often makes the biopsy difficult and risky, with reported pneumothorax rates of 4% to 27% and pulmonary hemorrhage rates of 0.3% to 4% of CT-guided percutaneous lung biopsy [21–23].

In retrospective series of SBRT studies, up to 65% of patients are treated without a pre-treatment pathological diagnosis of malignancy [24, 25]. In our report, 46% (55/119) of patients underwent HT-SBRT without a pathological diagnosis. The main reasons for the lack of pathological diagnosis were as follows: (1) 30 patients were not indicated for a biopsy procedure because of multiple medical complications, such as COPD, cardiovascular disease and abnormal coagulation function; (2) 20 patients refused a biopsy procedure even at the initial examination, although it was recommended by their physicians; (3) biopsy failed in 3 patients, and these patients refused re-biopsy; (4) 1 patient declined refused biopsy due to strong suspicion of newly developed second primary NSCLC that may be inoperable; and (5) 1 patient declined biopsy because the tumor was so small that there was little chance of confirming the pathology.

In our study, patients without pathological evidence achieved satisfactory outcomes, which was consistent with previous reports of SBRT for clinically diagnosed early-stage lung cancer [7–10]. The main finding of this study was that there was also no significant difference in LC, PFS, CSS, and OS between the clinically diagnosed and pathologically diagnosed cohorts, which is consistent [11, 13, 14, 26] or partially inconsistent [12, 27] with many previous studies.

Dautruche et al. [11] first performed a cohort analysis comparing clinically and pathologically based treatment for SBRT, which showed that no significant differences in patterns of recurrence, 3-year LC (85% vs. 80%, *p* = 0.78) and OS (71% vs. 74%, *p* = 0.48) after SBRT. A large retrospective study showed that there was no difference in the 3-year LC (91.2% vs. 90.4%, *p* = 0.98) and OS (55.4% vs. 53.7%, *p* = 0.99) between 209 patients with a clinical diagnosis and 382 NSCLC patients treated with SBRT [13]. Takeda et al. [14] studied 58 patients with a clinical diagnosis and 115 patients with a pathological diagnosis who were diagnosed as T1–4N0M0 (T1–T2a 92%) and treated with SBRT. No significant differences were observed between groups with or without pathological diagnosis in 3-year local control (80% vs. 87%, *p* = 0.73), progression-free survival (64% vs. 67%, *p* = 0.45), cancer-specific survival (74% vs. 71%, *p* = 0.57) or overall survival (54% vs. 57%, *p* = 0.48). An international multicenter study of 701 patients reported that there were no statistically significant differences in DFS (*p* = 0.64), CSS (*p* = 0.43), OS (*p* = 0.24) or recurrence between the no-biopsy and biopsy cohorts at a minimum of 2 years of comprehensive follow up[26]. Shaikh et al.[12]queried the Surveillance, Epidemiology, and End Results registry (SEER) database, including 6399 (90.8%) pathologically diagnosed and 651 (9.2%) clinically diagnosed patients and revealed that clinical diagnosis was associated with an improved CSS (HR = 0.82, 95% CI 0.71–0.96) but was not associated with an improved overall survival (HR = 1.01, 95% CI 0.90–1.13). A multicenter propensity score matching (PSM) study in a Chinese population showed similar results in terms of LC (*p* = 0.498) and OS (*p* = 0.141) between patients with and without a pathological diagnosis, while PFS (*p* = 0.008) was significantly better in the non-pathological group than the pathological group[27]. These disparities may be due to an increased likelihood of benign lesions in the clinical diagnosis cohort, which prevented them from developing cancer-related death or disease progression. The results of the previous studies are summarised in chronological order (Table 5).

**Table 5:** Reports of SBRT for clinically diagnosed early-stage lung cancer

Group	Study design	T-stage	Clinical diagnosis No. (%)	Pathological diagnosis No. (%)	Follow-up time (months)	Results (clinical diagnosis vs. pathological diagnosis)
Inoue et al. [7]	Retrospective	T1-2a	115 (100)	0 (0)	14	3-year OS: 89.8% (T1a), 60.7% (T1b/T2a); 5-year OS: 89.8% (T1a), 53.1% (T1b/T2a)
Verstegen et al. [12]	Retrospective	T1-2	382 (65)	209 (35)	29.5 and 32.8	3-year OS: 55.4% vs. 53.7% (*p* = 0.99); 3-year LC: 91.2% vs. 90.4% (*p* = 0.982)
Takeda et al. [14]	Retrospective	T1-4	58 (34)	115 (66)	20.2 and 21.2	3-year LC: 80% vs. 87% (*p* = 0.73), 3-year PFS: 64% vs. 67% (*p* = 0.45), 3-year CSS: 74% vs. 71% (*p* = 0.57), 3-year OS: 54% vs. 57% (*p* = 0.48)
Sakanaka et al. [9]	Retrospective	T1-2a	37 (100)	0 (0)	39	3-year LC: 95.2% (T1a) and 91.7% (T1b/T2a), 3-year OS: 89.7% (T1a) and 49.3% (T1b/T2a)
Harkenrider et al. [8]	Retrospective	T1-2	34 (100)	0 (0)	16.7	2-year regional control: 80%, 2-year distant control: 85%, 2-year OS: 85%
Yoshitake et al. [10]	Retrospective	T1-2a	88 (100)	0 (0)	23	3-year LC: 90%, 3-year PFS: 67%, 3-year OS: 80%
Shaikh et al. [12]	Retrospective	T1-2a	651 (9)	6399 (91)	17	5-year OS: 21.2% vs. 21% (*p* = 0.872), 5-year CSS: 48.8% vs. 38.1%(*p* = 0.013)
Dautruche et al. [11]	Retrospective matched-cohort analysis	T1-3	131 (50)	131 (50)	26	3-year LC:85% vs. 80% (*p* = 0.78), 3-year OS:71% vs. 74% (*p* = 0.48)
Fernandez et al. [26]	Multi-institutional retrospective	T1-3	248 (33)	504 (67)	44	5-year LC: 93.2% vs. 87.9% (*p* = 0.10), 5-year DFS: 43.9% vs. 46.5% (*p* = 0.64), 5-year CSS: 87.0% vs. 86.6% (*p* = 0.43), 5-year OS: 60.0% vs. 62.2% (*p* = 0.24)
Zhang et al. [27]	Multi-institutional Propensity-Matched Analysis	T1-2	45 (50)	45 (50)	58.3 and 56.3	5-year LC: 89.8% vs. 85.5% (p = 0.50), 5-year PFS: 70.9% vs. 40.6% (p = 0.008), 5-year OS: 76.1% vs. 63.2% (p = 0.14)
Fan et al. this study	Retrospective	T1-2	55 (46)	64 (54)	69	5-year LC: 87% vs. 83% (*p* = 0.58), 5-year PFS: 48% vs. 45% (*p* = 0.82), 5-year CSS: 87% vs. 84% (*p* = 0.65), 5-year OS: 60% vs. 63% (*p* = 0.79)

We also noted in our report that the downward trend in 5-year OS was more pronounced in patients with clinical diagnosis than in those with pathological diagnosis (60% vs. 63%), compared with results censored at 3 years (77% vs. 71%). With the extension of follow-up time, factors such as age and comorbidities may become more important for survival in patients with clinical diagnosis, which could also explain the lack of a significant decrease in cancer-specific survival over this period. In addition, few high-grade toxicities were observed in both cohorts of our study, which is consistent with previous studies. Analysis based on patients with T2 stage (> 3 cm) and high SUVmax (> 6) showed no statistically significant differences in LC and OS (*p* all > 0.05).

Although these excellent results strongly support the validity of SBRT in patients without a pathological diagnosis, clinical diagnosis has drawbacks that cannot be ignored. The first point is that a subset of neuroendocrine tumors with poor prognosis, such as small cell lung cancer (SCLC) and large cell neuroendocrine carcinoma (LCNEC), may be included in the clinical diagnosis. The local efficacy and safety of SBRT for SCLC and LCNEC have been reported in a number of publications [28–30]. However, because they tend to relapse at distant sites, their treatment usually requires local and systemic therapy. Therefore, treatment without pathological confirmation does not provide optimal systemic therapy such as chemotherapy for these patients with SCLC or LCNEC. Secondly, an absence of pathological confirmation may fail to provide information regarding prognostic factors such as epidermal growth factor receptor (EGFR) mutation in adenocarcinoma, which is also important in guiding adjuvant therapy [31]. Finally, the possibility of a benign lesion in clinical diagnosis cohorts cannot be ignored even if discussed by the MDT, which may lead to inadvertent overtreatment in this population.

With the use of radiotherapy for early-stage lung cancer, the total absolute number of patients without pathological diagnosis may increase in the future, so it is crucial to predict the likelihood of malignancy as accurately as possible. Swensen et al. [32] developed and validated the clinical prediction model consisting of factors such as age, smoking, history of extra-thoracic cancer, nodule diameter, location, and morphology. Herder et al. [33] integrated the findings of 18F-FDG PET imaging findings into the Swensen model to improve the accuracy of the combined model. Several new serum biomarkers under investigation are also being used to aid in the detection of early-stage lung cancer, such as plasma osteopontin (OPN) and circulating genetically abnormal cells (CACs) [34, 35]. In our study, we used a comprehensive prediction model that integrates clinical information, blood biomarker testing, and CT imaging results, which has previously been validated in a high-risk Chinese population and shown to have a sensitivity and specificity of nearly 90% in identifying lung cancer [36]. The application of these algorithms and novel biomarkers may help to rationally calculate the probability of malignancy and select patients for SBRT without a pathological diagnosis.

Some limitations of our study need to be mentioned when interpreting our results. First, a methodological limitation is that the present study was a single-institution retrospective study. Given the small sample size and the inherent risk of bias in retrospective analysis, we were unable to perform subgroup analyses to identify the clinical populations with greater benefit or no benefit from omitting biopsy. Therefore, a multi-institutional study with a large sample size and post-hoc subgroup analysis is recommended in the future. Second, there was a lack of information on subsequent adjuvant and salvage decisions, which also have a significant impact on survival outcomes. Third, quality of life data were not collected, although the survival and toxicity outcomes were reported.

## Conclusions

In conclusion, our results suggest that the efficacy and safety of clinically and pathologically diagnosed early-stage lung cancer treated with HT-SBRT in a multidisciplinary setting may be comparable. To our knowledge, this study is the longest reported follow-up of SBRT for patients without pathological diagnosis presented to date. It should be emphasized that the purpose of our study was not to encourage patients to omit biopsy, but to provide another option for patients with SPNs highly suggestive of malignancy when a definitive pathological diagnosis is not available/refused. We recommend that pathological confirmation should be attempted in all patients with suspected or clinically confirmed lung cancer prior to SBRT. For patients ineligible for biopsy, a validated comprehensive prediction model and various non-invasive or minimally invasive modalities including FDG-PET and liquid biopsy should be used in a multidisciplinary setting to estimate the likelihood of malignancy as accurately as possible. Individualized treatment and evidence-based management with appropriate follow-up protocols after SBRT are essential for patients with early-stage lung cancer without pathological diagnosis.

## Supplementary Information

**Additional file 1: Figure S1**. The flowchart of patient selection.

**Additional file 2: Table S1**. Probability of malignancy.

**Additional file 3: Figure S2**. Trends in receipt of clinical diagnosis (A) and distribution of diagnosis type stratified by time (B).

**Additional file 4: Figure S3**. Distribution of the calculated probability of malignancy for patients with either a clinical or pathological diagnosis.

**Additional file 5: Table S2**. Cause of death.

**Additional file 6: Figure S4**. Patterns of failure after SBRT for patients with clinical (A) and pathological diagnosis (B).

**Additional file 7: Table S3**. Patterns of failure.

## Data Availability

The datasets used and/or analyzed during the current study available from the corresponding authors on reasonable request.

## Abbreviations

BED_10_: Biologically effective dose using a/b-ratio of 10 Gy
CCI: Charlson comorbidity index
COPD: Chronic obstructive pulmonary disease
CSS: Cancer-specific survival
CT: Computed tomography
ECOG PS: Eastern Cooperative Oncology Group performance status
ES-NSCLC: Early-stage non-small cell lung cancer
FDG-PET: Fluorodeoxyglucose-positron emission tomography
GTV: Gross tumor volume
Gy: Gray
HR: Hazard ratio
HT: Helical tomotherapy
IMRT: Intensity-modulated radiotherapy
ITV: Internal target volume
LC: Local control
LCNEC: Large cell neuroendocrine carcinoma
MDT: Multidisciplinary team
OARs: Organs at risk
OS: Overall survival
PFS: Progression-free survival
PTV: Planning target volume
SBRT: Stereotactic body radiotherapy
SCLC: Small cell lung cancer
SPNs: Solitary pulmonary nodules
SUVmax: Maximal standardized uptake value

## References

[1] Guckenberger M, Andratschke N, Dieckmann K, Hoogeman MS, Hoyer M, Hurkmans C (2017). ESTRO ACROP consensus guideline on implementation and practice of stereotactic body radiotherapy for peripherally located early stage non-small cell lung cancer. Radiother Oncol.

[2] Videtic GMM, Donington J, Giuliani M, Heinzerling J, Karas TZ, Kelsey CR (2017). Stereotactic body radiation therapy for early-stage non-small cell lung cancer: Executive Summary of an ASTRO Evidence-Based Guideline. Pract Radiat Oncol.

[3] National Comprehensive Cancer Network. Non-small cell lung cancer. Version 4. 2021 [Internet]. 2021.

[4] Chi A, Jang SY, Welsh JS, Nguyen NP, Ong E, Gobar L (2011). Feasibility of helical tomotherapy in stereotactic body radiation therapy for centrally located early stage nonsmall-cell lung cancer or lung metastases. Int J Radiat Oncol Biol Phys.

[5] Casutt A, Bouchaab H, Beigelman-Aubry C, Bourhis J, Lovis A, Matzinger O (2015). Stereotactic body radiotherapy with helical TomoTherapy for medically inoperable early stage primary and second-primary non-small-cell lung neoplasm: 1-year outcome and toxicity analysis. Br J Radioly..

[6] X. Zheng, M. Schipper, K. Kidwell, J. Lin, R. Reddy, Y. Ren, et al. Survival outcome after stereotactic body radiation therapy and surgery for stage I non-small cell lung cancer: a meta-analysis. International journal of radiation oncology, biology, physics. Int J Radiat Oncol Biol Phys; 2014;90, 603-611. 10.1016/j.ijrobp.2014.05.055 25052562

[7] Inoue T, Shimizu S, Onimaru R, Takeda A, Onishi H, Nagata Y (2009). Clinical outcomes of stereotactic body radiotherapy for small lung lesions clinically diagnosed as primary lung cancer on radiologic examination. Int J Radiat Oncol Biol Phys.

[8] Harkenrider MM, Bertke MH, Dunlap NE (2014). Stereotactic body radiation therapy for unbiopsied early-stage lung cancer: a multi-institutional analysis. Am J Clin Oncol Cancer Clin Trials.

[9] Sakanaka K, Matsuo Y, Nagata Y, Maki S, Shibuya K, Norihisa Y (2014). Safety and effectiveness of stereotactic body radiotherapy for a clinically diagnosed primary stage I lung cancer without pathological confirmation. Int J Clin Oncol.

[10] Yoshitake T, Nakamura K, Shioyama Y, Sasaki T, Ohga S, Shinoto M (2015). Stereotactic body radiation therapy for primary lung cancers clinically diagnosed without pathological confirmation: a single-institution experience. Int J Clin Oncol.

[11] Dautruche A, Filion E, Mathieu D, Bahig H, Roberge D, Lambert L (2020). To biopsy or not to biopsy?: A matched cohort analysis of early-stage lung cancer treated with stereotactic radiation with or without histologic confirmation. Int J Radiat Oncol Biol Phys.

[12] Shaikh T, Churilla TM, Murphy CT, Zaorsky NG, Haber A, Hallman MA (2016). Absence of pathological proof of cancer associated with improved outcomes in early-stage lung cancer. J Thorac Oncol.

[13] Verstegen NE, Lagerwaard FJ, Haasbeek CJA, Slotman BJ, Senan S (2011). Outcomes of stereotactic ablative radiotherapy following a clinical diagnosis of stage I NSCLC: comparison with a contemporaneous cohort with pathologically proven disease. Radiother Oncol.

[14] Takeda A, Kunieda E, Sanuki N, Aoki Y, Oku Y, Handa H (2012). Stereotactic body radiotherapy (SBRT) for solitary pulmonary nodules clinically diagnosed as lung cancer with no pathological confirmation: Comparison with non-small-cell lung cancer. Lung Cancer.

[15] Amin MB, Edge SB, Greene FL (2017). AJCC cancer staging manual.

[16] He J, Huang Y, Shi S, Hu Y, Zeng Z (2015). Comparison of effects between central and peripheral stage I lung cancer using image-guided stereotactic body radiotherapy via helical tomotherapy. Technol Cancer Res Treat.

[17] Common Terminology Criteria for Adverse Events (CTCAE). Version 5.0. [Internet]. 2017. p. 155.

[18] Hansell DM, Bankier AA, MacMahon H, McLoud TC, Müller NL, Remy J (2008). Fleischner society: glossary of terms for thoracic imaging. Radiology.

[19] Schemper M, Smith TL (1996). A note on quantifying follow-up in studies of failure time. Control Clin Trials.

[20] Gould MK, Fletcher J, Iannettoni MD, Lynch WR, Midthun DE, Naidich DP (2007). Evaluation of patients with pulmonary nodules: when is it lung cancer?: ACCP evidence-based clinical practice guidelines (2nd edition). Chest.

[21] Chojniak R, Isberner RK, Viana LM, Yu LS, Aita AA, Soares FA (2006). Computed tomography guided needle biopsy: experience from 1,300 procedures. Sao Paulo Med J.

[22] Laspas F, Roussakis A, Efthimiadou R, Papaioannou D, Papadopoulos S, Andreou J (2008). Percutaneous CT-guided fine-needle aspiration of pulmonary lesions: Results and complications in 409 patients. J Med Imaging Radiat Oncol.

[23] Chakrabarti B, Earis JE, Pandey R, Jones Y, Slaven K, Amin S (2009). Risk assessment of pneumothorax and pulmonary haemorrhage complicating percutaneous co-axial cutting needle lung biopsy. Respir Med.

[24] Murray L, Ramasamy S, Lilley J, Snee M, Clarke K, Musunuru HB (2016). Stereotactic ablative radiotherapy (SABR) in patients with medically inoperable peripheral early stage lung cancer: outcomes for the first UK SABR cohort. Clin Oncol (R Coll Radiol).

[25] Lagerwaard FJ, Verstegen NE, Haasbeek CJA, Slotman BJ, Paul MA, Smit EF (2012). Outcomes of stereotactic ablative radiotherapy in patients with potentially operable stage I non-small cell lung cancer. Int J Radiat Oncol Biol Phys.

[26] Fernandez C, Grills IS, Ye H, Hope AJ, Guckenberger M, Mantel F (2020). Stereotactic Image guided lung radiation therapy for clinical early stage non-small cell lung cancer: a long-term report from a multi-institutional database of patients treated with or without a pathologic diagnosis. Pract Radiat Oncol.

[27] Zhang R, Guo Y, Yan Y, Liu Y, Zhu Y, Kang J (2021). A propensity-matched analysis of survival of clinically diagnosed early-stage lung cancer and biopsy-proven early-stage non-small cell lung cancer following stereotactic ablative radiotherapy. Front Oncol.

[28] Shioyama Y, Nakamura K, Sasaki T, Ohga S, Yoshitake T, Nonoshita T (2013). Clinical results of stereotactic body radiotherapy for Stage I small-cell lung cancer: a single institutional experience. J Radiat Res.

[29] Reshko LB, Kalman NS, Hugo GD, Weiss E (2018). Cardiac radiation dose distribution, cardiac events and mortality in early-stage lung cancer treated with stereotactic body radiation therapy (SBRT). J Thorac Dis.

[30] Lo H, Abel S, Finley G, Weksler B, Colonias A, Wegner RE (2020). Surgical resection versus stereotactic body radiation therapy in early stage bronchopulmonary large cell neuroendocrine carcinoma. Thorac Cancer.

[31] Lynch TJ, Bell DW, Sordella R, Gurubhagavatula S, Okimoto RA, Brannigan BW (2004). Activating mutations in the epidermal growth factor receptor underlying responsiveness of non-small-cell lung cancer to gefitinib. N Engl J Med.

[32] Swensen SJ, Silverstein MD, Edell ES, Trastek VF, Aughenbaugh GL, Ilstrup DM (1999). Solitary pulmonary nodules: clinical prediction model versus physicians. Mayo Clin Proc.

[33] Herder GJ, van Tinteren H, Golding RP, Kostense PJ, Comans EF, Smit EF (2005). Clinical prediction model to characterize pulmonary nodules: validation and added value of 18F-fluorodeoxyglucose positron emission tomography. Chest Chest.

[34] Blasberg JD, Pass HI, Goparaju CM, Flores RM, Lee S, Donington JS (2010). Reduction of elevated plasma osteopontin levels with resection of non-small-cell lung cancer. J Clin Oncol.

[35] Feng M, Ye X, Chen B, Zhang J, Lin M, Zhou H (2021). Detection of circulating genetically abnormal cells using 4-color fluorescence in situ hybridization for the early detection of lung cancer. J Cancer Res Clin Oncol.

[36] Yang D, Zhang X, Powell CA, Ni J, Wang B, Zhang J (2018). Probability of cancer in high-risk patients predicted by the protein-based lung cancer biomarker panel in China: LCBP study. Cancer.

