# Supplementary figures and images for: Comparison of long-term outcomes of stereotactic body radiotherapy (SBRT) via Helical tomotherapy for early-stage lung cancer with or without pathological proof
Source: Radiat Oncol. 2023 Mar 8;18:49. doi: 10.1186/s13014-023-02229-0 (PMC9996902; doi:10.1186/s13014-023-02229-0)

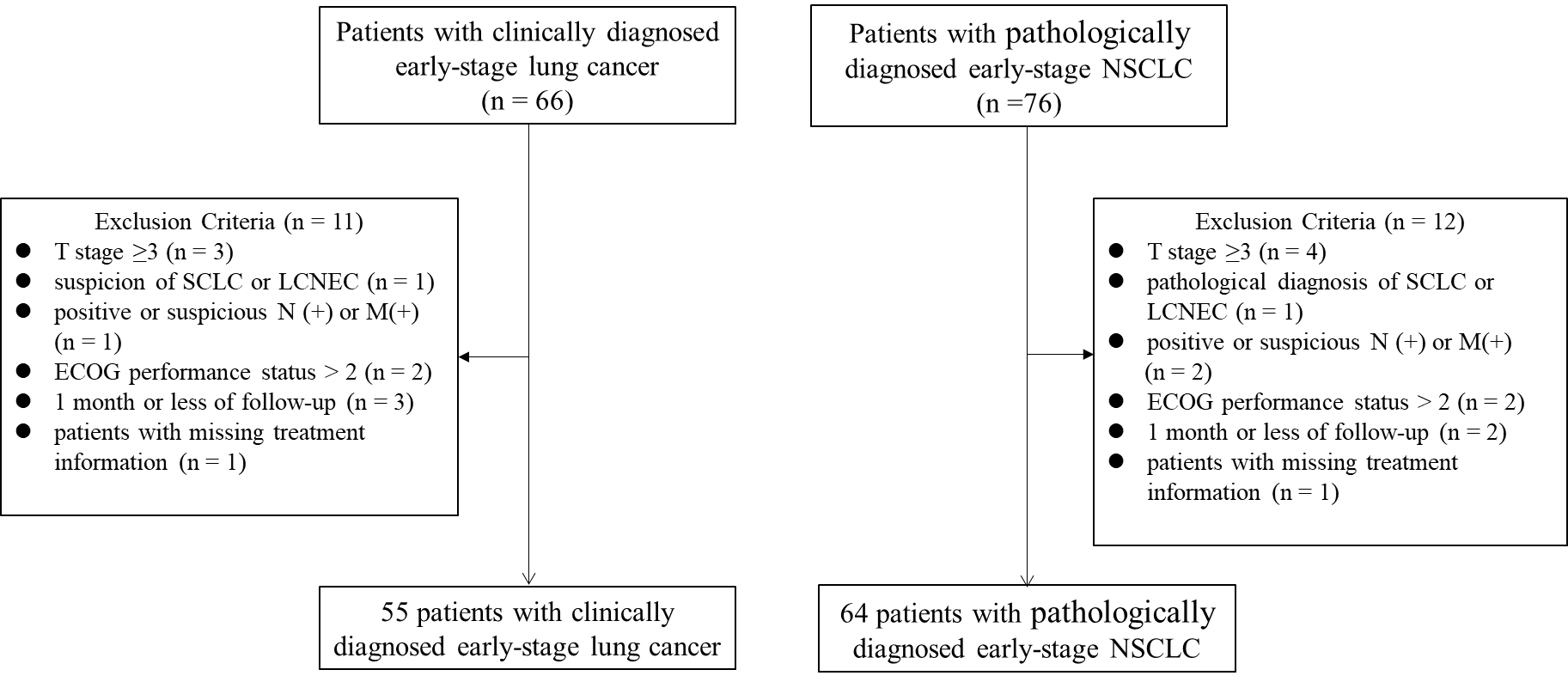

Supplement: Supplementary file 1 — Additional file 1: Figure S1. The flowchart of patient selection. [file 13014_2023_2229_MOESM1_ESM.tif]

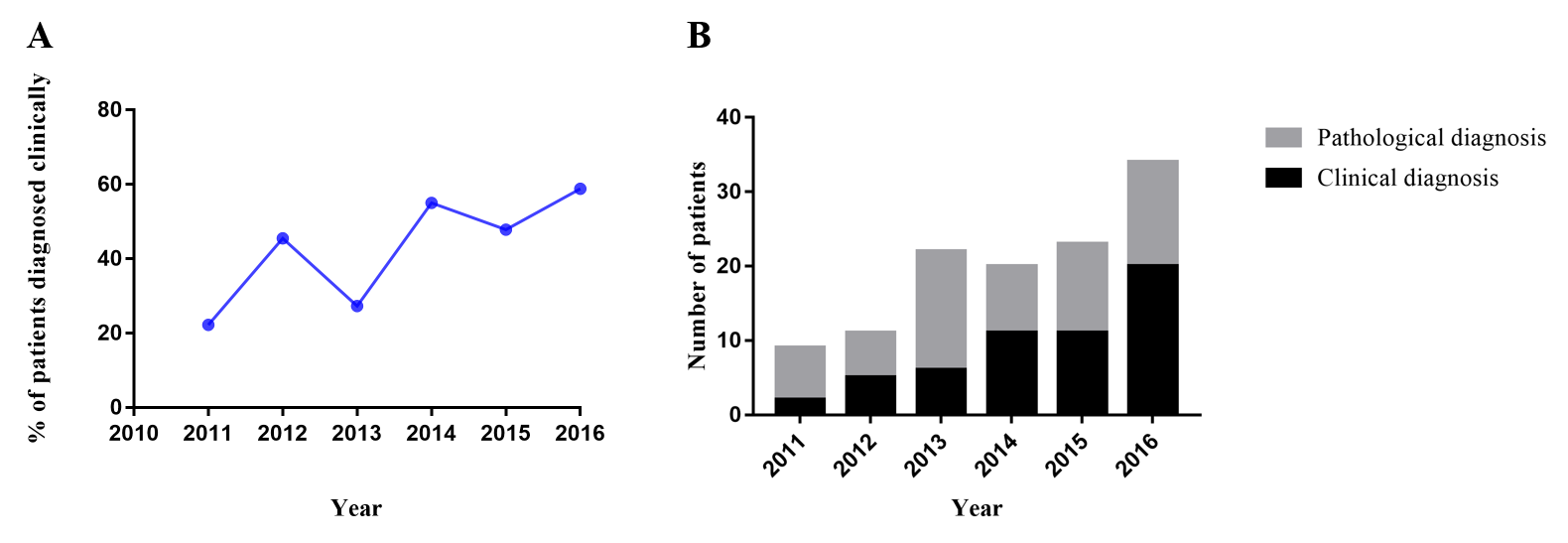

Supplement: Supplementary file 3 — Additional file 3: Figure S2. Trends in receipt of clinical diagnosis (A) and distribution of diagnosis type stratified by time (B). [file 13014_2023_2229_MOESM3_ESM.tif]

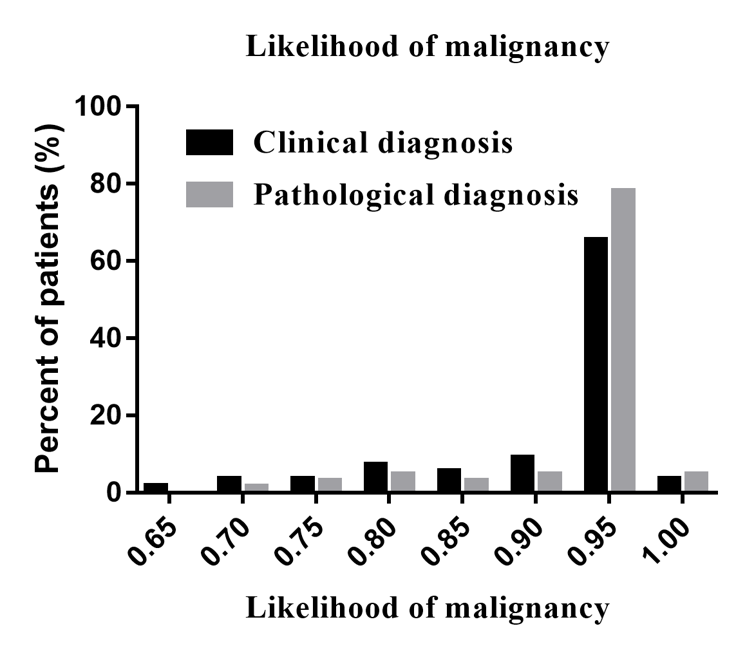

Supplement: Supplementary file 4 — Additional file 4: Figure S3. Distribution of the calculated probability of malignancy for patients with either a clinical or pathological diagnosis. [file 13014_2023_2229_MOESM4_ESM.tif]

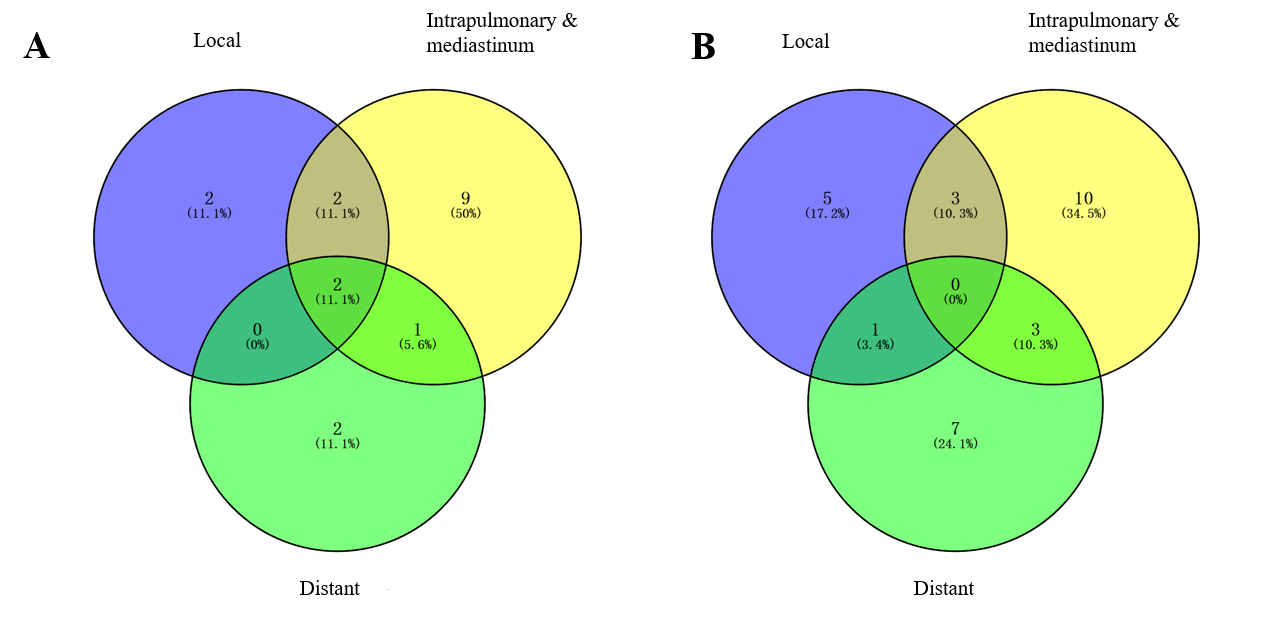

Supplement: Supplementary file 6 — Additional file 6: Figure S4. Patterns of failure after SBRT for patients with clinical (A) and pathological diagnosis (B). [file 13014_2023_2229_MOESM6_ESM.tif]
